# Evaluation of passenger satisfaction of urban multi-mode public transport

**DOI:** 10.1371/journal.pone.0241004

**Published:** 2020-10-20

**Authors:** Xinhuan Zhang, Hongjie Liu, Mingming Xu, Chengyuan Mao, Junqing Shi, Guolian Meng, Jinhong Wu

**Affiliations:** 1 The Institute of Road and Traffic Engineering, Zhejiang Normal University, Jinhua, Zhejiang Province, China; 2 School of Electronic and Information Engineering, Xi’an Jiao Tong University, Xi’an, Shanxi Province, China; 3 Ningbo Public Transport Administration Bureau, Ningbo, Zhejiang Province, China; University of Defence, SERBIA

## Abstract

The scientific evaluation of passenger satisfaction for public transport is helpful to enhance the attraction of public transport. To improve the accuracy of passenger satisfaction evaluation for public transport and the scientificity and objectivity of the index weighting, combining the characteristics of analytic hierarchy process (AHP), entropy weight method (EWM) and fuzzy comprehensive evaluation(FCE) method, the passenger satisfaction evaluation system for Ningbo’s urban public transportation was built. The paper analyzed 5046 questionnaires on conventional bus transit and 1682 questionnaires on rail transit in Ningbo city, Passenger satisfaction for Ningbo city’s public transport was evaluated comprehensively, and the evaluation results showed that the overall passenger satisfaction of the public transport in Ningbo was 91.2 in 2019, The case study shows that the application of the AHP-EWM-FCE model on the multi-mode public transport system can objectively quantify passengers’ feelings about urban public transport service, and thus provide a theoretical basis for the improvement of passenger satisfaction in Ningbo.

## I. Introduction

With the increase of urban population and the expansion of urban space, single-mode public transport can no longer meet the growth of travel demand. Multiple modes of public transport are constantly integrated into urban public transport networks, making urban public transport in a multi-mode state. As various modes of public transport are independent and influence each other, the evaluation and improvement of passenger satisfaction have become the focus of the traffic management department in such a multi-mode environment to attract passenger flow.

Passenger satisfaction of public transport service refers to a psychological state of satisfaction or disappointment after comparing the expectations of passengers about the services provided by the public transport system with their overall feelings after receiving the services [1], which can be expressed by the average score of the questionnaire on passenger satisfaction of public transport service during the survey period [2]. The passenger satisfaction survey, on the one hand, promotes the public’s participation in the improvement of the urban public transport system. On the other hand, it also promotes the public transport enterprises to grasp the core of the operation of the urban public transport system. The improvement (or deterioration) of public transport system for a city is usually reflected in the score of passenger satisfaction. Also, the degree of passenger satisfaction with different attributes of the public transportation system indicates the priority of improving the public transportation service.

In the process of evaluation of passenger satisfaction of urban public transportation, there are various types of evaluation indexes and they are related to each other. Fuzzy comprehensive evaluation (FCE) method is suitable for solving various non-deterministic problems, but it is not good at directly giving the weight of each index. The weights determined by AHP method reflect the subjective weights with expert intention to a great extent, while the objective weights calculated by EWM(entropy weight method) are inherently strong in mathematical theory. Combined with the characteristics of AHP and EWM, the complementary combination was realized, the comprehensive weight is more reasonable. It is more practical to use AHP- EWM(entropy weight method)-FCE model to evaluate passenger satisfaction.

To simplify the research content, in this paper, only the multi-mode public transport system composed of the two typical modes of conventional bus transit and rail transit is considered as the research object. Taking the multi-mode public transport in Ningbo as an example, the characteristics of the analytic hierarchy process(AHP), EWM(entropy weight method) and fuzzy comprehensive evaluation(FCE) methods was combined to build a passenger satisfaction evaluation system for urban public transportation. The application of the AHP-EWM-FCE model in the field of public transport can improve the scientificity and objectivity of index weighting, objectively quantify the passengers’ feelings towards the city’s public transport service, and help to propose improvement suggestions from the aspects of public transport operators and managers in the future, to provide the theoretical basis for the improvement of passenger satisfaction.

## II. Literature review

### A. Study on passenger satisfaction

The concept of customer satisfaction was first proposed by Richard (1965) [3], and subsequent studies have elaborated on the concept of customer satisfaction from different research perspectives. Many experts and scholars comprehensively used various methods to establish an evaluation model for measuring customer satisfaction. Early studies mainly included Oliver (1980) who established an expected inconsistency model [4], Fornell C., Larcker D. F. (1981) studied the structural equation model with latent variables and measurement errors [5], Churchillg. A. J r and Carol Surprenant’s (1982) cognitive performance model [6], Sasser’ s (1987) customer service level model [7], Engel and Blackwell’s (1993) weighted evaluation model [8]. The method of determining the weight is varied, it is particularly important to choose the right weight determination method; in general, the structural equation method (SEM) and driver measurement method have been widely applied [9].

The SERVQUAL (Service Quality) model was established in 1988 by three scholars, A. Prasuraman, Valarie A. Zeithaml and Leonard L.berry (PZB for short). The SERVQUAL model introduces regression analysis into the research and uses this method to assign the weight of customer satisfaction factors in data processing, which is of great significance to the study of customer satisfaction [10]. Sweden established the Sweden Customer Satisfaction Barometer in 1989, and the United States established the ACSI (American Customer Satisfaction Index) based on the Swedish Customer Satisfaction Barometer in 1994. After Sweden and the United States, the European Customer Satisfaction Index (ECSI) model has been established in Europe, and its internal structure has been innovated based on the research in the first two countries to make it develop continuously. New Zealand, Canada, South Korea and other countries have followed suit by creating their customer satisfaction measurement systems. In 2018, a new methodology for improving the measurement of the quality of the service consisting of three phases has been developed [11], the new methodology considers the assessment of the quality dimensions of a large number of participants (customers), on the one hand, and experts’ assessments on the other hand. The methodology was verified through the research carried out in an express post company. In 2019, optimal route criteria for Transport of hazardous material (THM) are selected using a new approach in the field of multi-criteria decision-making [12]. Weight coefficients of these criteria were determined by applying the Full Consistency Method (FUCOM). Evaluation and selection of suppliers is determined by applying the TOPSIS (Technique for Order of Preference by Similarity to Ideal Solution) and the MABAC (Multi-attributive Border Approximation Area Comparison) methods. The proposed route model was tested on the real example of the transport Eurodiesel in Serbia. When defining criteria that have an influence on traffic accessibility, Stanković, M [13] compare the significance of particular criteria using the Fuzzy AHP method and the Rough AHP method, which would show differences in the values of weight significance criteria and their ranking.

Ruisong Yu and Junxiang Cai (2012) proposed a "last kilometer" bus satisfaction evaluation model for Shanghai city by analyzing the operation characteristics of shuttle-bus lines in suburban areas and aiming at the difficulty of traveling in the "last kilometer" of public transport [14]. Jie Yu (2013) [15] focused on the study of conventional bus transit service in small and medium-sized cities, starting from the characteristics of conventional bus transit in small cities, he established a satisfaction evaluation index system for conventional bus transit service in small and medium-sized cities and studied it with the matter-element model. Yucheng Dong (2015) [16] took conventional bus transit of Lianyungang city as an example and constructed a service satisfaction evaluation index system by using WLS method to analyze factors such as bus running speed, ticket price, the attitude of personnel, in-car facilities and station density. Yakun Liu (2015) took 6 districts and counties in Urumqi city as research objects through a questionnaire survey, and conducted the satisfaction evaluation of conventional bus transit with the method of fuzzy evaluation [17]. Huanming Wang and Dajian Zhu (2010), combined with the performance evaluation of public transport services in Shanghai, proposed measures to improve the satisfaction of public transport services, including institutional and financial aspects [18]. Hongmei Wang and lingyu Jia (2011) used the matter-element model to analyze and evaluate the passenger satisfaction of public transport, specifically considering the core needs of passengers for public transport services [19].

### B. Empirical study of evaluation on public transport service

Friman et al. (2001) established an evaluation model to evaluate the customer satisfaction of public transport and concluded that the overall satisfaction was positively correlated with the cumulative satisfaction [20]. Kennedy et al. (2005) believed that the public should participate in public transport management and analyzed the influence of the public on the satisfaction of public transport [21]. Tyrinopoulos and Antoniou (2008) respectively used the factor analysis model and logit model to analyze passenger satisfaction with public transport performance [22]. Based on the survey data of South Africa, Mokonyama and Venter (2013) used the joint analysis model to identify the satisfaction levels of different service levels of the public [23]. Fiorio et al. (2013) used the survey data of 33 European cities in 2009 to analyze the correlation between public satisfaction and LPT (Local Public Transport), and found that the highest level of public satisfaction was associated with a single LPT provider [24]. Also, the researchers also studied and analyzed the passenger satisfaction of public transport services in Stockholm, Sweden [25], Bilbao, Spain [26], and Istanbul, Turkey [27]. In 2019, a new model that implies the integration of Full Consistency Method and a Rough Power Heronian aggregator for the selection of criteria for the quality of passenger service in rail transport, from the perspective of persons with disabilities as the main category of passengers, has been created [28]. The survey has covered 168 criteria classified in several groups and the entire territory of Serbia. Blagojević, A [29] In order to solve the criteria selection problem, the Fuzzy Analytical Hierarchical Processes (FAHP) method was experimented with, which showed the priority of the assessment of the efficiency of railway undertakings, on the basis of the five groups of criteria.

Fanghui Zheng (2005) took bus passengers as the investigation object in Guangzhou city, collected and sorted out the questionnaire, obtained the evaluation result of the passenger satisfaction of conventional bus transit, and proposed improvement measures based on the dissatisfaction factors such as the first and last shift, ticket price, and driving speed [30]. Guobing Hu, Sun, etc. (2011) [31] uses the four points graph model, and, from the perspective of passengers, determines the score of evaluation index and its weights, finds the root cause of passenger dissatisfaction and puts forward related suggestions, the feasibility, effectiveness, and extensibility of the index system, the analysis method and the related suggestions are proved by the case study of public transport in Nanchang city. Xiuzhen Guo and Xiaoxiong Weng (2014) [32] used the analytic hierarchy process (AHP) to determine the weight of indicators, and evaluated the level of public transport service with the method of fuzzy comprehensive evaluation. The AHP method represents a formal framework for solving complex multiatributive decision making problems, as well as a systemic procedure for ranking multiple alternatives and/or for selecting the best from a set of available ones [33]. In reference to the American customer satisfaction index (ACSI) model, based on the quality of service, service facilities, service, safety, environment and other four aspects, Lili Jiao (2012) [34] established the index system, built the customer satisfaction index model of urban rail transit of China, and used partial least square method to estimate the model, with the practicability of the model verified by an example.

From the above research status, it can be seen that studies on customer satisfaction are mostly based on practical cases, and different countries have their satisfaction measurement models. Most scholars concentrated on the study of satisfaction factors, models, and the construction of an evaluation index system, focusing on how to build a satisfaction evaluation model for a single-mode public transport, such as bus lines and bus transfers in different cities and carry out case verification. Each study has a certain theoretical and practical value. However, there are still deficiencies in theoretical and practical studies, which are mainly reflected in the following two aspects:

First, the current literature on passenger satisfaction of urban public transport service is not comprehensive enough, and the indicator system needs to be further improved. In particular, the analysis of factors affecting passenger satisfaction of public transport service is not thorough enough in terms of passengers’ demands for comfort, convenience, waiting time, and accessibility.

Second, the existing research is limited to the single-mode evaluation of the passenger satisfaction: urban conventional bus transit or rail transit, and pays less attention to the overall satisfaction evaluation of urban public transport that includes multiple modes.

## III. Methodology

The research involves completing a questionnaire to evaluate passenger satisfaction, in this questionnaire, if the participants are interested in this research, they will be asked to leave travel information about urban multi-mode public transportation of Ningbo city. All the participation in the project/survey is entirely voluntary and the participant are free to withdraw from the project at any point without giving reason. Any information and data were collected and analyzed anonymously.

In the process of passenger satisfaction evaluation of public transport, there are many evaluation indexes and they are related to each other. The fuzzy comprehensive evaluation(FCE) method is based on the fuzzy set theory developed by Zadeh [35] for capturing the uncertainties inherent in a system. The fuzzy evaluation approach can provide a powerful mathematical tool to quantify imprecise information in human judgments. But since it is not good at directly giving the weight of each evaluation index, combining it with the AHP method can improve the objectivity of index weighting.

The AHP method has been generally accepted as a powerful multi-criteria decision-making tool for dealing with complex decision problems in public transport research domains. In this paper, the AHP method has been used to determine the weights of different indexes during the evaluation process based on expert judgments. In later sections it will be shown how this method can be coupled with a fuzzy approach to enhance its ability to capture the uncertainties and vagueness of satisfaction perceptions expressed by the passengers. It is more practical and reasonable to use AHP-FCE to evaluate passenger satisfaction of the multi-mode public transport.

**Fig 1:**
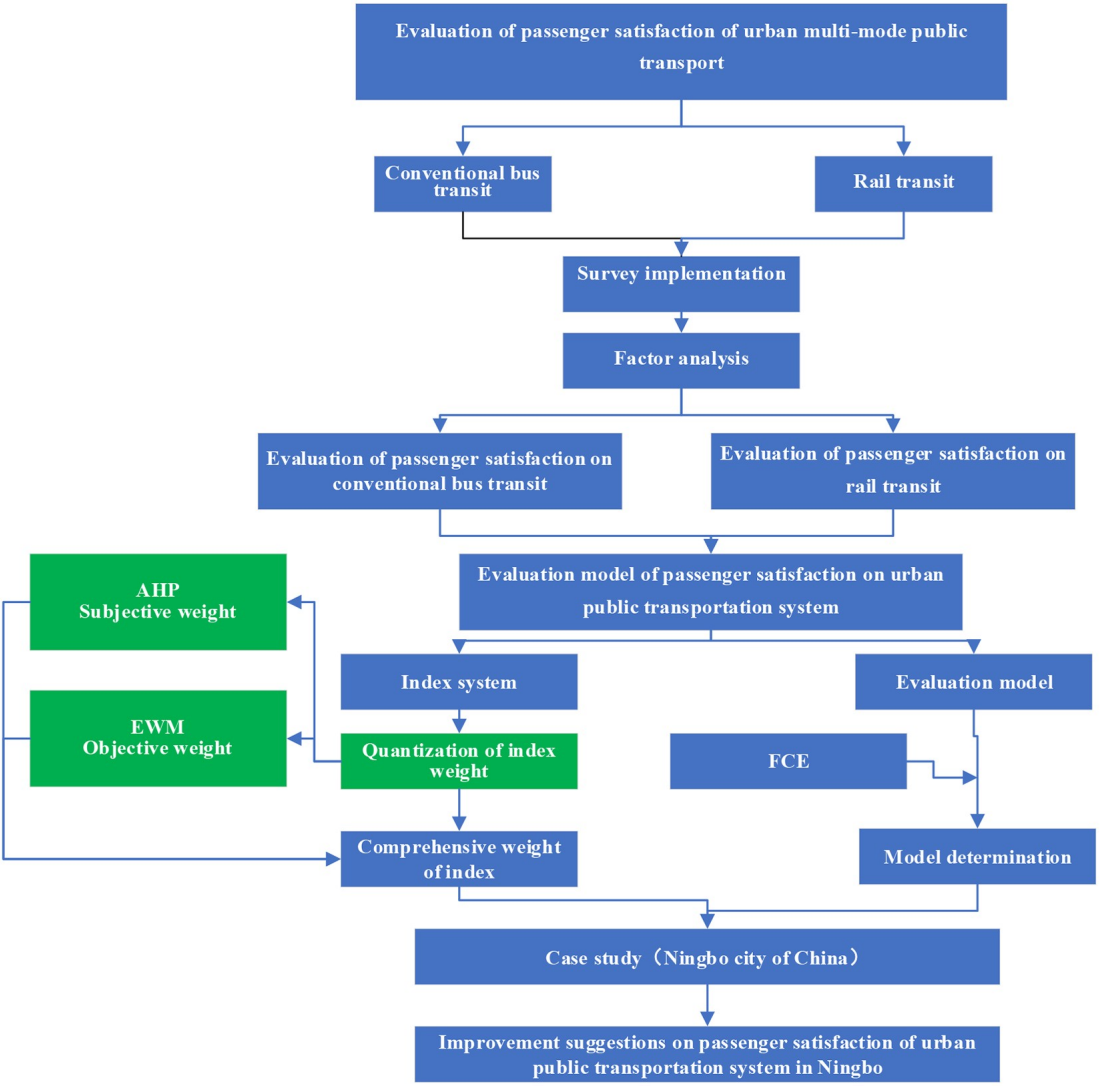
Flow chart of research methods.

The research procedure of this paper is as follows (Fig 1).

### A. Determining the weight vector by AHP

Based on the actual data obtained from the questionnaire survey, this paper evaluates the annual urban multi-model public transport service in Ningbo city and puts forward corresponding countermeasures and suggestions according to the problems shown in the evaluation results.

Based on the AHP method, the weight vector *W*_*si*_ (see step 3 in subsection A) determined by conducting empirical and/or field studies of transportation professionals reflects the intention of decision-makers, which is the subjective weight from consulting expert opinions. One advantage of the AHP method is that it provides both an elicitation method as well as a strong theoretical framework that allows precise quantitative calculations. The procedures of the AHP method can be illustrated step by step as follows:

**Step 1:** Structure a hierarchy of the criteria based on the evaluated factors.
  Passenger satisfaction of public transport is obtained under the influence of multi-dimensional psychological factors, and the factors are related to each other in a complex way. A hierarchical evaluation model is established based on the principle of giving priority to passengers:
  First, state an overall objective for the problem and list factors that affect the objective. In this case, evaluation target O = {passenger satisfaction with urban public transport service}, according to the evaluation object O, the evaluation criteria set B = {B1, B2 …, Bn}and sub-criteria set C = {C1, C2 …, Cn}. In the case of this paper, B = {waiting time B1, transfer convenience B2, service B3, information B4, passenger comfort B5, station environment B6, interior sanitation B7}. Then structure a hierarchy of criteria for the problem: for each cluster or level in the hierarchy, some factors will be subjected to a corresponding evaluated objective.
**Step 2:** Construct a pairwise comparison matrix.
  The major advantage of the AHP method is that, instead of asking experts to directly give a weight for a particular evaluation factor, they will be asked to rate the relative importance of the different factors. The expert group composed of transportation professionals, through the process of integration, communication, and feedback, uses scaling law proposed by Thomas L. Saaty [36], assuming that there are n evaluation factors, the importance intensity of factor i over factor j can be represented by Aij. A complete pairwise comparison matrix A can, therefore, be obtained.
**Step 3:** Calculate the priority vectors of evaluated factors.
  To calculate the weight vectors of evaluated factors, we used the common method of ANC (average of normalized columns). ANC can be presented as:
  $$W_{si}=\frac{1}{n}\sum_{j=1}^{n}\frac{A_{ij}}{\sum_{i=1}^{n}A_{ij}}(i,j=1,2,\ldots ,n)$$ (1)
  The weight vector can therefore be obtained from matrix A by normalizing the vector in each column and then averaging over the rows of the resulting matrix.
  The weight determined based on the AHP method reflects the intention of decision-makers, so it is a subjective weight: *W*_*si*_ is the subjective weight. Determine the weight vector of the evaluation index, and make the weight distribution set Wi to the index set B or C is *W*_*si*_, then the subjective weight set of all levels of the index is W = {*W*_*s*1_, *W*_*s*2…_, *W*_*sn*_}.
**Step 4:** Check the consistency of human judgments (consistency check of single-sorting).
  From Step 3, the numerical subjective weights = {*W*_*s*1_, *W*_*s*2…_, *W*_*sn*_} of the factors from the numerical judgments matrix A can be obtained. It is important to check that the human judgments are internally consistent. One method is to calculate the consistency ratio (CR)to reduce the possibility of consistent random deviation, the maximum eigenvalue λ_*max*_ and the consistency index CI of the judgment matrix are calculated of the matrix, then calculate the consistency ratio CR which is a measure of how a given matrix compares to a purely random matrix in terms of their consistency indices:
  $$CI=\frac{1}{n-1}(\lambda_{max}-n)$$ (2)
  CR=CI/RI (3)
  Where, RI is the average random index, which is computed and tabulated as shown in Tables [36], If a value of the consistency ratio CR <0.1, the numerical judgments will be considered to be acceptable [36], and the comparison matrix corresponding to the hierarchical index has passed the consistency check. Otherwise, it is necessary to readjust the index value of the matrix to achieve consistency.
**Step 5:** Check the consistency of human judgments(consistency check of total-sorting).
  The total-sorting of all levels refers to the sorting weight value of the indexes of each level relative to the indexes of the highest level. Assuming that there are n elements in k layer, when CR(k)<0.1, the whole judgment matrix is considered to pass the consistency check.

### B. Determining the comprehensive weight vector by AHP-EWM

The basic idea of the entropy weight method (EWM) is to determine the objective weight according to the index variability. Compared with various subjective weighting models, the biggest advantage of the EWM is the avoidance of the interference of human factors on the weight of indicators, thus enhancing the objectivity of the comprehensive evaluation results [37,38]. Generally speaking, the smaller the information entropy of an index is, the greater the degree of variation of the index value will be, the more information it provides, the greater the role it can play in the comprehensive evaluation, and the greater its weight will be, and vice versa.

The weight determined based on the AHP method reflects the intention of decision-makers, so it is a subjective weight. However, the weight determined by the entropy weight method does not consider the intention of decision-makers, but has a strong mathematical theoretical basis and purely reflects the relationship between data. The two kinds of weights have some limitations, so they are combined organically to give a comprehensive weight that reflects both objective information and subjective information.

Information entropy can be used to measure the amount of information. The indexes under each criterion layer form an evaluation matrix. EWM calculates each weight according to the evaluation matrix, which reflects the influence of the index data itself on the weight in the objective information of evaluation, and is an objective weight.

Formulas are as follows:

$$P_{ij}=\frac{x_{ij}}{\sum_{i=1}^{m}x_{ij}}(i=1,2,\ldots \ldots ,m;j=1,2,\ldots ,n)$$ (4)

$$e_{j}=-k\sum_{i=1}^{m}P_{ij}lnP_{ij}(j=1,2,\ldots ,n),k\geq 0,e_{j}\geq 0$$ (5)

$$W_{oi=}\frac{g_{j}}{\sum_{j=1}^{n}g_{j}}(j=1,2,\ldots ,n)$$ (6)

Where, *x*_*ij*_ is the value of the jth index of the ith sample; *P*_*ij*_ is the proportion of the ith sample of the jth index; *e*_*j*_ is the entropy value of the jth index, and k is related to the number of samples. Generally, let k = 1/ lnm, and 0<e<1. *W*_*oi*_ is the objective weight of the jth index; *g*_*j*_ is the differential coefficient of the jth index, and. *g*_*j*_ = 1 − *e*_*j*_ (*j* = 1,2,…, *n*).

Combining the subjective and objective information, determine the comprehensive weight, that is:

$$W_{=}\frac{W_{si}∙W_{oi}}{\sum_{i=1}^{n}W_{si}∙W_{oi}}$$ (7)

Where, *W* is the combined weight, *W*_*si*_, *W*_*oi*_ respectively is the subjective weight and the objective weight. The weights of the criteria and sub-criteria in the case are obtained by formula (7).

### C. Comprehensive evaluation of passenger satisfaction based on FCE

**Step 1:**Determining the set of evaluation factors.
  Evaluation factors can be defined according to the objectives of the satisfaction evaluation process. A set of n evaluation factors can be represented as a vector C = {C1, C2, …., Cn}. For example, one can define C = {C1, C2, …., C20} = {waiting time, punctuality of first and last shift. …, Sanitary facilities in the vehicle}such that different measurements will be conducted to evaluate the public transport service based on these 20 factors.
**Step 2:**Determining the set of appraisal grades.
  The evaluation criteria of each evaluation index are divided into 4 grades, namely: satisfaction, comparative satisfaction, basic satisfaction and dissatisfaction, and these grades are assigned to 100, 80, 60 and 0, so the evaluation set V = {V1,V2,V3,V4} = = {100,80,60,0}.
**Step 3:** The questionnaire survey.
  The actual FCE questionnaire was generated based on the evaluation indexes of the hierarchical model. For conventional bus transit and rail transit, passengers were asked to fill in the FCE questionnaire. The FCE questionnaire was collected and preliminary survey results were analyzed.
**Step 4:** Setting the fuzzy mapping matrix.
  The goal of the evaluation process is to provide a mapping from C to V. For a specific factor Ci, the fuzzy mapping to the appraisal vector V can be represented by the vector R_i_ = {r_i1_,  r_i2_, …, r_ik_, …, r_im_}, in which m represents the number of levels in the appraisal (see step 2), and r_ik_ represents the fuzzy membership degree of appraisal factor i to grade k. In the Ningbo city case, single factor evaluation of the single factor Ci(i = 1,2,…,n) was made in the indicator set C, and then according to the proportion of people with grade V_m_ in the total people in the ith index, the single factor evaluation set of the ith factor is obtained, and the evaluation membership matrix R is obtained: *r*_*i*_ = (*r*_*i*1_, *r*_*i*2_, Λ, *r*_*nm*_). In general, the fuzzy appraisal matrix of all n factors can be derived and represented as a matrix R, such that if there are n factors and m levels of appraisal grades:
  $$R=\left[\begin{array}{cccc} r_{11} & r_{12} & \Lambda & r_{1m}\\ r_{21} & r_{22} & \Lambda & r_{2m}\\ Μ & Μ & Ο & Μ\\ r_{n1} & r_{n2} & \Lambda & r_{nm} \end{array}\right]$$
  In the above matrix notation for R, each row represents the set of appraisal membership degrees to the corresponding appraisal vector V for each evaluation factor C_i_ in the evaluation vector C.
  Take the overall evaluation result B, that is, the membership vector of the evaluation index element set to the evaluation grade set.
  $$B=W_{C}\cdot R=(W_{C1},W_{C2},\Lambda ,W_{Cn})\cdot [\begin{array}{llll} r_{11} & r_{12} & \Lambda & r_{1m}\\ r_{21} & r_{22} & \Lambda & r_{1m}\\ \text{Μ} & \text{Μ} & \text{O} & \text{Μ}\\ r_{n1} & r_{n2} & \Lambda & r_{nm} \end{array}]={\left(B_{1}^{*},B_{2}^{*},\Lambda ,B_{n}^{*}\right)}^{T}$$
**Step 5:** Determining the weight of each evaluation factor.
  To obtain a comprehensive passenger satisfaction evaluation, the relative importance of each evaluation factor on the overall grading of public transport should be quantified. The weight vector can be represented by W, which can be formulated by the AHP method, as described in the A subsection. As above, for n evaluation factors, the weight can be represented by the vector W = (W_1_, W_2_, …, W_n_), in which the sum of all elements equal 1. From the example discussed later, if it is determined that W_B_ = (0.22, 0.24, 0.15, 0.14, 0.08, 0.08, 0.09), then the relative weights satisfaction will be (0.22, 0.24, 0.15, 0.14, 0.08, 0.08, 0.09) for B = {waiting time B1, transfer convenience B2, service B3, information B4, passenger comfort B5, station environment B6, interior sanitation B7} respectively.
**Step 6:** Getting the overall appraisal result.
  The overall appraisal result can be obtained by considering the relative weights of each evaluation factor, such that a single vector with the same level of appraisal grades m (see step 2) can be represented by:
  O=A∘V=(A1,A2,…,An)∘V
  Where ‘∘’ is a composition operator.
  According to the questionnaire results, the weights of evaluation indexes determined by AHP-EWM and the scores of each grade, the comprehensive evaluation results of conventional bus transit and rail transit were calculated respectively. Take the conventional but transit for example:
  $${\text{O}}_{\text{Conventional}}=A\circ \text{V}=(A1,A\;2,\ldots ,A\text{n})\circ \text{V}$$
  $$=[\begin{array}{cccc} 0.57 & 0.33 & 0.09 & 0.02 \end{array}]\times [\begin{array}{c} 100\\ 80\\ 60\\ 0 \end{array}]=88.32$$
**Step 7:** Synthesizing the result vector of fuzzy synthetic evaluation.
  The comprehensive passenger satisfaction evaluation on public transport in the whole city was obtained by combined the evaluate results of conventional bus and rail transit. The formula is as follows:
  $$\begin{array}{l} \begin{array}{l} \text{O}=((\text{the}\;\text{average}\;\text{score}\;\text{of}\;\text{a}\;\text{single}\;\text{questionnaire}\;\text{for}\;\text{conventional}\;\text{bus}\;\text{transit}\\ {}*\text{the}\;\text{number}\;\text{of}\;\text{questionnaires}\;\text{for}\;\text{conventional}\;\text{bus}\;\text{transit}) \end{array}\\ \begin{array}{l} +(\text{the}\;\text{average}\;\text{score}\;\text{of}\;\text{a}\;\text{single}\;\text{questionnaire}\;\text{for}\;\text{rail}\;\text{transit}))\\ {}*\text{the}\;\text{number}\;\text{of}\;\text{questionnaires}\;\text{for}\;\text{rail}\;\text{transit}) \end{array}\\ \begin{array}{l} /(\text{the}\;\text{number}\;\text{of}\;\text{questionnaires}\;\text{for}\;\text{conventional}\;\text{bus}\;\text{transit}\\ +\text{the}\;\text{number}\;\text{of}\;\text{questionnaires}\;\text{for}\;\text{rail}\;\text{transit}) \end{array} \end{array}$$

## IV. Data preprocessing

### A. Overview

This paper takes public transport of Ningbo city as a case to verify and the research scope mainly includes the conventional bus transit and rail transit services in Haishu District, Jiangbei District, Beilun District, Zhenhai District, and Yinzhou District. The research objective is to evaluate the passenger satisfaction with public transport service in Ningbo in 2019.

In 2019, 7 conventional bus transit companies were operating in the five Districts of Ningbo, with 6,484 operating vehicles and 1,250 routes. There are 1,140 standard vehicles in operation on rail lines 1, 2, and 3, with a total number of 456 vehicles and 76 groups. The total length of the lines in operation is 91 kilometers. There are 66 stations on the lines, and the rated capacity of passengers is 110,960.

### B. The data processing

#### 1) The sampling rate

According to statistics released on December 30, 2019, the population of the central urban areas of Ningbo (Haishu District, Jiangbei District, Yinzhou District, Beilun District, and Zhenhai District) actually totaled 4.11 million. According to the proportion of 3 parts per 10,000 required by the evaluation index of public transport city, it is calculated that the number of sampling samples should reach more than 1233.

According to the evaluation requirement of "the proportion of questionnaire issuance between conventional bus transit and rail transit shall be distributed according to the proportion of passenger traffic" in cities, conventional bus transit accounts for about 75% of the total passenger traffic of public transport (conventional bus transit and rail transit), and rail transit accounts for about 25% of the total passenger traffic of public transport (conventional bus transit and rail transit). The number of rail transit questionnaires matched with the number of 5046 valid conventional bus transit questionnaires was about 1682.

#### 2) Survey implementation

The cycle of passenger satisfaction evaluation of public transport in the downtown area of Ningbo in 2019 is from January to December. The survey is conducted in the following two ways:

1. Conventional bus transit.
  Ningbo south railway station, Gulou bus station, Ningbo passenger transport center station, Youngor Stadium station, Dongmen station, Baisha central station, Gaoqiao station, Donghuan south road station, etc.
2. Rail transit:
  Stations of line 1: Gaoqiao station, Daqingqiao station, Gulou station, Dongmen station, Sakura park station, Fuqing north road station.
  Stations of line 2: Yinzhou Avenue station, Light textile city station, Passenger transport center station, Railway station, Bund bridge station, Lulin station.

After eliminating waste, abnormal and incomplete volumes, this survey obtained 6728 valid questionnaires, including 5046 for conventional bus transit and 1682 for rail transit. The proportion of indicator C_i_ choosing V_n_ in the questionnaire is shown in Tables 1 and 2.

**Table 1:** Passenger evaluation of index of passenger satisfaction of conventional bus transit.

Criteria	Sub-criteria	Happy 100	Satisfied 80	Basically satisfied 60	Not satisfied 0
B1 waiting time	C1 waiting time	35.45%	43.29%	17.17%	4.09%
	C2 punctuality of first and last shift	46.95%	40.53%	11.12%	1.40%
B2 transfer convenience	C3 route setting	62.40%	29.76%	5.79%	2.05%
	C4 transfer convenience between buses	77.13%	19.12%	3.42%	0.33%
	C5 transfer convenience between bus transit and rail transit	58.50%	34.12%	6.50%	0.88%
	C6 transfer convenience between bus and bicycle	56.64%	34.63%	7.49%	1.24%
B3 service	C7 station voice announcement in-bus	71.75%	24.37%	3.58%	0.30%
	C8 service attitude of the crew	65.59%	28.54%	5.48%	0.39%
	C9 air conditioning	67.60%	26.51%	5.38%	0.51%
	C10 discounts for seniors, students, and IC cards	80.40%	16.93%	2.44%	0.23%
B4 information	C11 sign definition of the station	67.21%	27.83%	4.50%	0.46%
	C12 the logo inside the vehicle	65.69%	28.60%	5.21%	0.50%
	C13 information query	59.77%	31.78%	7.74%	0.71%
	C14 recharge convenience for IC card	53.33%	30.97%	12.96%	2.74%
B5 passenger comfort	C15 performance of the vehicle, safety of seat and armrest	54.13%	37.19%	8.25%	0.43%
	C16 Congestion in during peak hours	22.38%	40.37%	28.06%	9.19%
B6 station environment	C17 waiting facilities of intermediate station	49.60%	38.37%	11.16%	0.87%
	C18 facilities of the terminal (departure station)	53.70%	35.73%	9.86%	0.71%
B7 interior sanitation	C19 interior hygiene	61.82%	30.42%	7.51%	0.25%
	C20 Sanitary facilities in the vehicle	61.47%	30.35%	7.58%	0.60%

**Table 2:** Passenger evaluation of index of passenger satisfaction of rail transit.

Criteria	Sub-criteria	Happy 100	Satisfied 80	Basically satisfied 60	Not satisfied 0
B’1 waiting time	C’1 waiting time	69.54%	24.97%	3.76%	1.73%
	C’2 punctuality of first and last shift	74.97%	20.47%	3.70%	0.86%
B’2 transfer convenience	C’3 the time of the last train	67.26%	23.18%	7.03%	2.53%
	C’4 frequency during rush hour	63.26%	28.18%	7.09%	1.48%
	C’5 transfer convenience between bus transit and rail transit	65.44%	26.85%	5.75%	1.96%
	C’6 transfer convenience between rail and bicycle	66.28%	27.74%	4.69%	1.29%
B’3 service	C’7 transfer convenience between rail transit and private car	59.37%	28.85%	8.51%	3.27%
	C’8 service attitude of the clerk	73.86%	21.45%	4.44%	0.25%
	C’9 convenience of using the automatic ticket machine	75.96%	19.85%	3.82%	0.37%
	C’10 layout of the check-in machine of the station	73.61%	22.07%	3.95%	0.37%
B’4 information	C’11 informing passengers about emergencies	72.44%	23.00%	4.38%	0.18%
	C’12 information service and the layout guide signs	74.66%	21.27%	3.70%	0.37%
	C’13 information query	71.45%	22.07%	5.86%	0.62%
	C’14 calls quality of mobile phone	81.13%	15.23%	3.58%	0.06%
B’5 passenger comfort	C’15 interior air and the comfort of air conditioner, seat	72.94%	20.66%	5.54%	0.86%
	C’16 Congestion in during peak hours	31.13%	40.69%	23.06%	5.12%
B’6 station environment	C’17 accessibility and cleanliness of the station	79.78%	15.47%	4.50%	0.25%
	C’18 facilities of the terminal (departure station)	82.49%	13.13%	3.70%	0.68%
B’7 interior sanitation	C’19 interior hygiene	83.05%	14.55%	2.28%	0.12%
	C’20 aesthetics of the internal environment	79.84%	16.77%	3.02%	0.37%

#### 3) Basic characteristics

1. Conventional bus transit.
  A total of 5,046 valid questionnaires were obtained from passenger sampling survey of conventional bus transit, among which 4,144 were carried out on the bus, with an average of 3 samples for each bus line. The station survey is 902 samples.
  - Gender: the percentage of the male and the female was 46.32% and 53.68% respectively among the bus passengers surveyed this year.
  - Age: young people aged between 16 and 25 years old account for 38% of the investigated passengers, which is mainly because young passengers are often enthusiastic participants in the evaluation of bus service. Between 26 and 35 years old, about 23%; between 36 and 45 years old, about 13%; between 46 and 65 years old, about 13%; about 6% were under 15 years old and 7% were over 65 years old.
  - Frequency: according to the survey results, the average number of bus rides per week varies greatly among the residents surveyed, with 0~4 times accounting for 29.17%; 5~9 times accounting for 26.55%; 10~15 times accounting for 24.23%. More than 15 times accounted for 20.05%.
2. Rail transit.
  The effective samples of the 2019 survey on rail transit in Ningbo were 1,682.
  - Gender: the male and the female accounted for 48% and 52% of the total sample, respectively.
  - Age: in terms of age distribution, passengers are mainly between 16 and 30 years old in this year’s survey, accounting for 73% of the total sample. Between 31 and 45 years old were about 15%, 46–65 years old were about 6%, and 5% were under 16 years old. About 1% are over 65 years old.
  - Frequency: among the surveyed passengers, those who take rail transit more than 10 times a week account for 28%, those who take it more than 6 to 10 times a week account for 20%, those who take it 3–5 times a week account for 29%, and those who take it 0–2 times a week account for 23%.

## V. Case study

### A. Evaluation model

For the multi-mode public transport of Ningbo city, the passenger satisfaction model was established from the perspective of people, vehicles, environment, and facilities and equipment used most frequently, to determine the passenger satisfaction evaluation index system of public transport service in Ningbo city in 2019.

### B. Hierarchical evaluation index matrix and consistency check

The results of the consistency check for both single-sorting and total-sorting of the comparison matrix for each level of indexes were calculated. According to formula (7), the weights of the criteria and sub-criteria of conventional bus transit and rail transit can be calculated and obtained, as shown in Table 3.

**Table 3:** Consistency check of the overall sorting of public transport in Ningbo city.

Conventional bus transit	The consistency check value of layer B to layer A CR = 0.040<0, and the consistency check is passed							
		B1	B2	B3	B4	B5	B6	B7
	Criterion weight W_B_	0.2186	0.239	0.1467	0.1433	0.0828	0.0822	0.0873
	CR	0	0.025	0.025	0.077	0	0	0
Rail transit	The consistency check value of layer B to layer A’ CR = 0.040<0, and the consistency check is passed							
		B '1	B '2	B '3	B '4	B '5	B '6	B '7
	Criterion weight W’_B’_	0.1016	0.2201	0.1429	0.1658	0.1996	0.0778	0.0921
	CR	0.025	0.089	0.089	0.03	0.03	0	0

#### 1) Conventional bus transit

Through the expert scoring of public transport professionals to the indicators in Ningbo city, consistent opinions were obtained after many times, the results of the statistics were in the comparison matrix, and comprehensive check indicators of the overall sorting are:

CR=(0.0239×0.0229+0.1467×0.0228+0.1433×0.0697)/(0.0239×0.9+0.1467×0.9+0.1433×0.9)=0.040<0.1

Therefore, the overall sorting of passenger satisfaction of conventional bus transit in Ningbo passed the consistency check.

According to formula (7), the weight of criteria and the weight of sub-criteria of the comprehensive index system for conventional bus transit can be calculated, as shown in Table 4.

**Table 4:** Overall sorting and weight calculation of conventional bus transit.

W_Ci_ weight	B1	B2	B3	B4	B5	B6	B7	comprehensive weights	sorting
	0.2186	0.2390	0.1467	0.1433	0.0828	0.0822	0.0873		
C1	0.5							0.1093	1
C2	0.5							0.1093	2
C3		0.1418						0.0339	17
C4		0.3290						0.0786	3
C5		0.3290						0.0786	4
C6		0.2002						0.0478	6
C7			0.2877					0.0422	10
C8			0.2470					0.0362	15
C9			0.1756					0.0258	20
C10			0.2887					0.0424	9
C11				0.3444				0.0494	5
C12				0.1972				0.0283	19
C13				0.2472				0.0354	16
C14				0.2111				0.0302	18
C15					0.5			0.0414	11
C16					0.5			0.0414	12
C17						0.5		0.0411	13
C18						0.5		0.0411	14
C19							0.5	0.0437	7
C20							0.5	0.0437	8

#### 2) Rail transit

The comprehensive check indicators of the total sorting is:

$$CR=\frac{0.1016\times 0.0227+0.2201\times 0.0516+0.1429\times 0.0517+0.1658\times 0.0176+0.1996\times 0.0176}{0.1016\times 0.9+0.2201\times 0.58+0.1429\times 0.58+0.1658\times 0.58+0.1996\times 0.58}=0.053<0.1$$

In the same way, unanimous opinions can be obtained through expert scoring. According to formula (7), the weight of the criteria and the weight of the sub-criteria of the comprehensive index system for rail transit can be calculated, as shown in Table 5.

**Table 5:** Total sorting and weight calculation of rail transit.

W_C’I_ weight	B '1	B '2	B '3	B '4	B '5	B '6	B '7	comprehensive weights	sorting
	0.1016	0.2201	0.1429	0.1658	0.1996	0.0778	0.0921		
C '1	0.2048							0.0208	19
C '2	0.1690							0.0172	20
C '3	0.2881							0.0292	17
C '4	0.3381							0.0344	16
C '5		0.4111						0.0905	3
C '6		0.2611						0.0575	6
C '7		0.3278						0.0722	4
C '8			0.4905					0.0701	5
C'9			0.3119					0.0446	10
C '10			0.1976					0.0282	18
C '11				0.2106				0.0349	15
C '12				0.5485				0.0909	2
C '13				0.2409				0.0399	12
C '14					0.2409			0.0481	7
C '15					0.5485			0.1095	1
C '16					0.2106			0.0420	11
C '17						0.5		0.0389	13
C '18						0.5		0.0389	14
C '19							0.5	0.0461	8
C '20							0.5	0.0461	9

### C. Fuzzy comprehensive evaluation

#### 1) Conventional bus transit

1. First-level fuzzy comprehensive evaluation.
  According to the questionnaire, the membership matrix R_1_, R_2_, R_3_, R_4_, R_5_, R_6_, and R_7_ of the fuzzy comprehensive evaluation of level 1 of the conventional bus transit are calculated as follows:
  $$R_{1}=\left[\begin{array}{cccc} 0.3545 & 0.4329 & 0.1717 & 0.0409\\ 0.4695 & 0.4053 & 0.1112 & 0.0140 \end{array}\right]$$
  $$R_{2}=\left[\begin{array}{cccc} 0.6240 & 0.2976 & 0.0579 & 0.0205\\ 0.7713 & 0.1912 & 0.0342 & 0.0033\\ 0.5850 & 0.3412 & 0.0650 & 0.0088\\ 0.5664 & 0.3463 & 0.0749 & 0.0124 \end{array}\right]$$
  $$R_{3}=\left[\begin{array}{cccc} 0.7175 & 0.2437 & 0.0358 & 0.0030\\ 0.6559 & 0.2854 & 0.0548 & 0.0039\\ 0.6760 & 0.2651 & 0.0538 & 0.0051\\ 0.8040 & 0.1693 & 0.0244 & 0.0023 \end{array}\right]$$
  $$R_{4}=\left[\begin{array}{cccc} 0.6721 & 0.2783 & 0.0450 & 0.0046\\ 0.6569 & 0.2860 & 0.0521 & 0.0050\\ 0.5977 & 0.3178 & 0.0774 & 0.0071\\ 0.5333 & 0.3097 & 0.1296 & 0.0274 \end{array}\right]$$
  $$R_{5}=\left[\begin{array}{cccc} 0.5413 & 0.3719 & 0.0825 & 0.0043\\ 0.2238 & 0.4037 & 0.2806 & 0.0919 \end{array}\right]$$
  $$R_{6}=\left[\begin{array}{cccc} 0.4960 & 0.3837 & 0.1116 & 0.0087\\ 0.5370 & 0.3573 & 0.0986 & 0.0071 \end{array}\right]$$
  $$R_{7}=\left[\begin{array}{cccc} 0.6182 & 0.3042 & 0.0751 & 0.0025\\ 0.6147 & 0.3035 & 0.0758 & 0.0060 \end{array}\right]$$
  Then, after the normalization of the membership vector of the first-lever fuzzy comprehensive evaluation, it is obtained.
  $$\begin{array}{cc} B_{1}^{*}= & {\text{W}}_{\text{C}}\cdot {\text{R}}_{1}=\left[\begin{array}{cc} 0.5 & 0.5 \end{array}\right] \end{array}\cdot \left[\begin{array}{cccc} 0.3545 & 0.4329 & 0.1717 & 0.0409\\ 0.4695 & 0.4053 & 0.1112 & 0.0140 \end{array}\right]=(0.41,0.42,0.14,0.03);$$
  $${\text{B}}_{2}^{*}=(0.65,0.29,0.06,0.01);$$
  $${\text{B}}_{3}^{*}=(0.72,0.24,0.04,0.00);$$
  $${\text{B}}_{4}^{*}=(0.62,0.30,0.07,0.01);$$
  $${\text{B}}_{5}^{*}=(0.38,0.39,0.18,0.05);$$
  $${\text{B}}_{6}^{*}=(0.52,0.37,0.11,0.00);$$
  $${\text{B}}_{7}^{*}=(0.62,0.30,0.08,0.00).$$
2. Second-level fuzzy comprehensive evaluation.
  The passenger satisfaction evaluation of conventional bus transit of Ningbo city is calculated as follows:
  $$\text{A}={\text{W}}_{\text{B}}\cdot \text{R}={\text{W}}_{B}\cdot \left[\begin{array}{c} B_{1}^{*}\\ B_{2}^{*}\\ B_{3}^{*}\\ B_{4}^{*}\\ B_{5}^{*}\\ B_{6}^{*}\\ B_{7}^{*} \end{array}\right]={\left[\begin{array}{c} 0.22\\ 0.24\\ 0.15\\ 0.14\\ 0.08\\ 0.08\\ 0.09 \end{array}\right]}^{T}\cdot \left[\begin{array}{cccc} 0.41 & 0.42 & 0.14 & 0.03\\ 0.64 & 0.29 & 0.06 & 0.01\\ 0.72 & 0.24 & 0.04 & 0.00\\ 0.62 & 0.30 & 0.07 & 0.01\\ 0.38 & 0.39 & 0.18 & 0.05\\ 0.52 & 0.37 & 0.11 & 0.00\\ 0.62 & 0.30 & 0.08 & 0.00 \end{array}\right]=(0.57,0.33,0.09,0.02)$$
3. The result vector of fuzzy comprehensive evaluation.
  According to the principle of weighted average to obtain the membership degree, the scores of each criterion layer of passenger satisfaction evaluation of the conventional bus transit are as follows:
  $${\text{O}}_{\text{B}1*}=B_{1}^{*}\times \text{V}=\left[\begin{array}{cccc} 0.41 & 0.42 & 0.14 & 0.03 \end{array}\right]∙\left[\begin{array}{c} 100\\ 80\\ 60\\ 0 \end{array}\right]=83.22$$
  O_B2*_ = 91.10, O_B3*_ = 93.32, O_B4*_ = 90.16, O_B5*_ = 80.17, O_B6*_ = 87.60, O_B7*_ = 90.48, The final score of passenger satisfaction evaluation of conventional bus transit is:
  $${\text{O}}_{\text{conventional}}=\text{A}\times \text{V}=\left[\begin{array}{cccc} 0.57 & 0.33 & 0.09 & 0.02 \end{array}\right]\left[\begin{array}{c} 100\\ 80\\ 60\\ 0 \end{array}\right]=88.32.$$

#### 2) Rail transit

Similarly, the evaluation of passenger satisfaction of rail transit of Ningbo can be calculated as follows:

$$\text{A}\prime ={\text{W}}_{\text{B}}\prime \cdot \text{R}\prime =\text{WB}\prime \cdot \left[\begin{array}{c} B\prime_{1}^{*}\\ B\prime_{2}^{*}\\ B\prime_{3}^{*}\\ B\prime_{4}^{*}\\ B\prime_{5}^{*}\\ B\prime_{6}^{*}\\ B\prime_{7}^{*} \end{array}\right]={\left[\begin{array}{c} 0.10\\ 0.22\\ 0.14\\ 0.17\\ 0.20\\ 0.08\\ 0.09 \end{array}\right]}^{T}\cdot \left[\begin{array}{cccc} 0.68 & 0.25 & 0.06 & 0.02\\ 0.64 & 0.28 & 0.06 & 0.02\\ 0.74 & 0.21 & 0.04 & 0.00\\ 0.73 & 0.22 & 0.04 & 0.00\\ 0.66 & 0.24 & 0.09 & 0.02\\ 0.81 & 0.14 & 0.04 & 0.00\\ 0.81 & 0.16 & 0.03 & 0.00 \end{array}\right]=(0.71,0.23,0.06,0.01)$$

According to the principle of weighted average to obtain the membership degree, the scores of each criterion layer of passenger satisfaction evaluation of rail transit are respectively

$${\text{O}}_{\text{B}’1*}=B\prime_{1}^{*}\times \text{V}=\left[0.68,0.25,0.06,0.02\right]∙\left[\begin{array}{c} 100\\ 80\\ 60\\ 0 \end{array}\right]=90.99$$

O_B’2*_ = 89.69, O_B’3*_ = 93.81, O_B’4*_ = 93.50, O_B’5*_ = 90.22,O_B’6*_ = 95.04, O_B7*_ = 95.56, The final score of passenger satisfaction evaluation of rail transit is:

$${\text{O}}_{{}_{\text{Rail}}}=\text{A}\prime \times \text{V}=\left[0.71,0.23,0.06,0.01\right]\times \left[\begin{array}{c} 100\\ 80\\ 60\\ 0 \end{array}\right]=92.10$$

#### 3) The overall evaluation

Based on the analysis of 5046 valid questionnaires of conventional bus transit and 1682 valid questionnaires of rail transit, according to the weight of each index, the satisfaction of conventional bus transit is calculated to be 88.32, and that of rail transit is 92.10. Passenger satisfaction of multi-mode public transport of Ningbo city is the comprehensive score of conventional bus transit and rail transit, and the calculation formula is as follows:

$$\begin{array}{l} {\text{O}}_{\text{Passenger}\;\text{satisfaction}\;\text{of}\;\text{public}\;\text{transport}\;\text{service}\;\text{of}\;\text{Ningbo}\;\text{city}}=((\text{the}\;\text{average}\;\text{score}\;\text{of}\;\text{a}\;\text{single}\;\text{questionnaire}\;\text{for}\;\text{conventional}\;\text{bus}\;\text{transit}\\ {}*\text{the}\;\text{number}\;\text{of}\;\text{questionnaires}\;\text{for}\;\text{conventional}\;\text{bus}\;\text{transit})\\ +(\text{the}\;\text{average}\;\text{score}\;\text{of}\;\text{a}\;\text{single}\;\text{questionnaire}\;\text{for}\;\text{rail}\;\text{transit})\\ {}*\text{the}\;\text{number}\;\text{of}\;\text{questionnaires}\;\text{for}\;\text{rail}\;\text{transit})\\ /(\text{the}\;\text{number}\;\text{of}\;\text{questionnaires}\;\text{for}\;\text{conventional}\;\text{bus}\;\text{transit}+\text{the}\;\text{number}\;\text{of}\;\text{questionnaires}\;\text{for}\;\text{rail}\;\text{transit})\\ =(88.32*5046+92.10*1682)/(5046+1682)=89.27 \end{array}$$

Therefore, it is calculated that the passenger satisfaction of multi-mode public transport in Ningbo city in 2019 is 89.27.

### D. Analysis of evaluation results

#### 1) Conventional bus transit

The evaluation results show that the evaluation of comfort is the worst, with the main problem being congestion during peak hours. The second is the waiting time, with the waiting time of the station and the punctuality of the first and last shift still far from the requirements of passengers; then it is the problem of setting up the waiting environment and bus station. In these seven aspects of the evaluation, the rating of driver service satisfaction is the highest, indicating that the work style of drivers is satisfactory to passengers currently.

In short, the riding comfort and punctuality evaluation of public transport is poor in rush hour. Therefore, the first step is to increase the frequency of buses during peak periods to reduce congestion and improve the comfort of taking buses. Secondly, it is necessary to make efforts in the punctuality of public transportation, which is mainly reflected in the scientific scheduling of vehicles, the speed in operation, and the smoothness of the road. Then, we should strengthen the setting of the bus stations and optimization of routes to improve the convenience of taking buses.

#### 2) Rail transit

The survey results show that in the satisfaction evaluation of the quality of phone calls, the staff’s response to inquiries and complaints are lower among the service indexes of each station. Also, the evaluation of the lighting facilities of Gaoqiao station and Daqingqiao station at the west of line 1 is relatively poor.

### E. Suggestions for improvement

From the analysis of the results, two suggestions on multi-mode public transport in Ningbo city are given:

#### 1) Optimization of bus station and network

To optimize the lines of conventional bus transit, we should combine the rail network, conventional bus transit network, bicycle network, pedestrian network and various transfer hubs, and scientifically constructing the multi-mode public transport structure, to minimize the transfer time and space. At the same time, the stations should be set with the aim of the convenience of the transfer and high service capacity of different passenger groups.

#### 2) Trials of speed up vehicle operation and schedule

At present, the main bottleneck in which the bus attraction is not high is that the bus is relatively poor on time, time consumption is too long on the road. Under the premise of giving priority to the right of way, speeding up the vehicles is an important measure to reduce the time consumption on the road, and improve the punctuality of public transport.

Also, it is suggested that some routes can carry out the pilot project of providing schedules for the intermediate stations in order to improve the punctuality of buses on the road. The bus lane can be set up, including the arterials and the collectors. Then adjust the transfer nodes of bus lines, and add and adjust supplementary facilities such as exclusive lanes and traffic lights to optimize the bus dispatching area.

## VI. Conclusion

Based on the analysis of 5046 valid questionnaires of conventional bus transit and 1682 valid questionnaires of rail transit, according to the weight of each index, the passenger satisfaction of multi-mode public transport service in Ningbo city is calculated to be 89.27, which includes the satisfaction of conventional bus transit at 88.32 and the rail transit at 92.10.

The research results show that the AHP- EWM model is one of the quantitative methods about index on passenger satisfaction of urban multimode public transportation system. AHP- EWM-FCE model is feasible in passenger satisfaction evaluation on multimodal public transportation system in Ningbo, which has certain application value. It aims to provide a certain reference for relevant public transportation companies and to make the operation and service evaluation of public transportation system more efficient.

But the AHP-EWM-FCE model is founded that there are still some shortage in the application process, the model is built on the basis of the analytic hierarchy process (AHP) and fuzzy comprehensive evaluation(FCE) methods, so there are still problems with the two methods applied in the process of itself. the EWM (entropy weight method) is adopted to modify the index weight, avoid the subjectivity of evaluation results to some extent, but the AHP- EWM-FCE model was only applied on Ningbo city, “the comprehensive evaluation can be applied in other cities?” also need to continuously be tested and improved in practice. In the follow-up research, more attention should be paid to the improvement of this model and the integration of passenger satisfaction of different modes on urban public transportation system, so as to make a better overall evaluation on urban multimode public transportation system.

## Supporting information

S1 File (DOCX)

S2 File (DOCX)
