# Supplementary material for: Evaluation of passenger satisfaction of urban multi-mode public transport
Source: PLoS One. 2020 Oct 20;15(10):e0241004. doi: 10.1371/journal.pone.0241004 (PMC7575119; doi:10.1371/journal.pone.0241004)
Supplement: S1 File — (DOCX) [file pone.0241004.s001.docx]

**宁波市城市公共交通乘客满意度调查问卷----常规公交**

尊敬的女士∕先生您好：

我们是浙江师范大学城市交通规划研究人员。本调查目的是为了推进“宁波公交都市”的建设。感谢您在百忙之中抽出时间填写这份问卷。本调查采用无记名方式，我们将严格遵守国家《保密法》的有关规定。谢谢您的帮助！

浙江师范大学工学院

**填表说明：请在您认为合适的序号前打“√”，问卷无特殊说明，为单选。**

**一、基本情况**

1、当前您乘坐的公交车线路：______

2、性别： ①男； ②女；

3、年龄： ① 15岁以下；②16-25岁；③26-35岁；④36-45岁；⑤46-65岁；⑥65岁以上；

4、职业是： ①学生；②工人；③职员；④公务员；⑤教师；⑥个体经营者；⑦农业劳动者；⑧军人；⑨离退休人员；⑩其它；

5、您平日主要的出行方式： ①公共汽车；②轨道交通；③出租车；④小汽车；⑤电瓶车；⑥自行车；⑦步行；

6、您本次出行目的：①上班；②上学；③公务；④生活购物；⑤文娱体育；⑥探亲访友；⑦看病；⑧回家；

7、您平均每星期乘坐公交车的次数为**：** ①15次以上；②10-15次； ③5-9次；④0-4次；

8、您目前月工资收入： ①<1650元； ②1651-2500元； ③2501-4077元； ④4078-6000元； ⑤6001-10000元； ⑥>10000元；

9、您家庭拥有车辆情况 ：①有多辆小汽车； ②有一辆小汽车； ③助动车； ④自行车；⑤无车；

**二、候车时间长度**

10、您平时在车站候车时间大约为：

① 0-5分钟；② 6-10分钟；③11-15分钟；④16-20分钟；⑤20分钟以上；

11、您对候车时间的满意程度： ①满意；② 比较满意；③合格；④不满意；

12、您对首、末班车准时性的满意程度： ①满意；② 比较满意；③合格；④不满意；

**三、换乘便捷度**

13、您对当前公交线路设置感到： ①方便；② 比较方便；③合格；④不方便；

14、您对当前公交与公交换乘便捷程度感到： ①方便；② 比较方便；③合格；④不方便；

15、您对当前公交与轨道交通换乘便捷程度感到：

①方便；② 比较方便；③合格；④不方便；

16、您对当前公交与公共自行车换乘便捷程度感到：

①方便；② 比较方便；③合格；④不方便；

**四、服务态度**

17、您对车内语音报站感到： ①满意；② 比较满意；③合格；④不满意；

18、您对司乘人员服务态度感到： ①满意；② 比较满意；③合格；④不满意；

19、您对空调车辆是否按规定使用空调感到： ①满意；② 比较满意；③合格；④不满意；

20、您对对老人和学生及IC卡票价优惠感到： ①满意；② 比较满意；③合格；④不满意；

**五、出行信息服务**

21、您对车站站牌清晰度感到： ①满意；② 比较满意；③合格；④不满意；

22、您对车内的标识(如行车路线、警示标志、服务和投诉电话等)的醒目程度感到：

①满意；② 比较满意；③合格；④不满意；

23、您对公交信息服务(网上、手机APP上公交信息查询) 感到：

①满意；② 比较满意；③合格；④不满意；

24、您对公交IC卡充值方便程度感到： ①满意；② 比较满意；③合格；④不满意；

**六、乘车舒适度**

25、您对车辆的新旧性能及车辆座椅、扶手的安全性感到：

①满意；② 比较满意；③合格；④不满意；

26、您感觉高峰时段车内拥挤程度： ①不拥挤；②轻度拥挤；③过得去；④重度拥挤

**七、候车环境**

27、您对中间停靠站候车设施感到： ①满意；② 比较满意；③合格；④不满意；

28、您对起终点站候车设施、便民设施感到： ①满意；② 比较满意；③合格；④不满意；

**八、车内卫生环境**

29、您对车内的卫生情况： ①满意；② 比较满意；③合格；④不满意；

30、您对车内的卫生设施配置： ①满意；② 比较满意；③合格；④不满意；

**编号____________________调查员___________________**

**宁波市城市公共交通乘客满意度调查问卷----轨道交通**

尊敬的女士∕先生您好：

我们是浙江师范大学城市交通规划研究人员。本调查目的是为了推进“宁波公交都市”的建设。感谢您在百忙之中抽出时间填写这份问卷。本调查采用无记名方式，我们将严格遵守国家《保密法》的有关规定。谢谢您的帮助！

浙江师范大学工学院

**填表说明：请在您认为合适的序号前打“√”，问卷无特殊说明，为单选。**

**一、基本情况**

1、当前您乘坐的轨道交通线路：______

2、性别： ①男； ②女；

3、年龄： ① 15岁以下；②16-25岁；③26-35岁；④36-45岁；⑤46-65岁；⑥65岁以上；

4、职业是： ①学生；②工人；③职员；④公务员；⑤教师；⑥个体经营者；⑦农业劳动者；⑧军人；⑨离退休人员；⑩其它；

5、您平日主要的出行方式： ①公共汽车；②轨道交通；③出租车；④小汽车；⑤电瓶车；⑥自行车；⑦步行；

6、您本次出行目的：①上班；②上学；③公务；④生活购物；⑤文娱体育；⑥探亲访友；⑦看病；⑧回家；

7、您平均每星期乘坐轨道交通的次数为**：** ①15次以上；②10-15次； ③5-9次；④0-4次；

8、您目前月工资收入： ①<1650元； ②1651-2500元； ③2501-4077元； ④4078-6000元； ⑤6001-10000元； ⑥>10000元；

9、您家庭拥有车辆情况 ：①有多辆小汽车； ②有一辆小汽车； ③助动车； ④自行车；⑤无车；

**二、候车时间长度**

10、您平时在车站候车时间大约为：

① 0-5分钟；② 6-10分钟；③11-15分钟；④16-20分钟；⑤20分钟以上；

11、您对候车时间感到： ①满意；② 比较满意；③合格；④不满意；

12、您对首、末班车准时性感到： ①满意；② 比较满意；③合格；④不满意；

**三、换乘便捷度**

13、您对当前轨道交通末班列车时间感到：

①方便；② 比较方便；③合格；④不方便；

14、您对当前轨道交通高峰时间的轨道交通班次感到：

①方便；② 比较方便；③合格；④不方便；

15、您对当前轨道交通与常规公交换乘便捷程度感到：

①方便；② 比较方便；③合格；④不方便；

16、您对当前轨道交通与公共自行车换乘便捷程度感到：

①方便；② 比较方便；③合格；④不方便；

**四、轨道交通服务质量**

17、您对当前轨道交通与私家车换乘便捷程度感到：

①满意；② 比较满意；③合格；④不满意；

18、您对轨道交通工作人员的主动服务及工作人员对问讯、咨询、投诉等响应感到： ①满意；② 比较满意；③合格；④不满意；

19、您对自动售票机使用方便性感到： ①满意；② 比较满意；③合格；④不满意；

20、您对进出站检票机布局感到： ①满意；② 比较满意；③合格；④不满意；

**五、出行信息服务**

21、您对突发情况告知乘客及时性感到：

①满意；② 比较满意；③合格；④不满意；

22、您对轨道交通信息服务及引导标识布局感到：

①满意；② 比较满意；③合格；④不满意；

23、您对轨道交通信息服务(网上、手机APP信息查询) 感到：

①满意；② 比较满意；③合格；④不满意；

24、您对轨道交通内手机通话质量感到：

①满意；② 比较满意；③合格；④不满意；

**六、乘车舒适度**

25、您对车内空气、空调舒适度及车辆座椅舒适性感到：

①满意；② 比较满意；③合格；④不满意；

26、您感觉高峰时段车内拥挤程度： ①不拥挤；②轻度拥挤；③过得去；④重度拥挤

**七、站厅环境**

27、您对站内畅通性与清洁卫生感到： ①满意；② 比较满意；③合格；④不满意；

28、您对起终点站候车设施、便民设施感到： ①满意；② 比较满意；③合格；④不满意；

**八、车内卫生环境**

29、您对列车内的清洁卫生： ①满意；② 比较满意；③合格；④不满意；

30、您对列车内环境的美观度： ①满意；② 比较满意；③合格；④不满意；

**编号____________________调查员___________________**
