# Supplementary material for: Evaluation of passenger satisfaction of urban multi-mode public transport
Source: PLoS One. 2020 Oct 20;15(10):e0241004. doi: 10.1371/journal.pone.0241004 (PMC7575119; doi:10.1371/journal.pone.0241004)
Supplement: S2 File — (DOCX) [file pone.0241004.s002.docx]

**Passenger Satisfaction Questionnaire of public Transport System in Ningbo City – Conventional bus transit**

Dear Madam/Sir,

We are researchers of urban traffic planning in Zhejiang Normal University, the purpose of this survey is to promote the construction of "Ningbo transit city". Thank you for taking time out of your busy schedule to fill in this questionnaire. The investigation will be conducted anonymously, and we will strictly abide by the relevant provisions of the State Secrets Law. Thank you for your help!

College of Engineering, Zhejiang Normal University

**Instructions: Please tick the serial number you think is appropriate. The questionnaire is single choice without special instructions.**

1. **Basic information**

1. Your current route line:________

2. Gender: (1) Male; (2) female;

3. Age: (1) under 15 years old; (2) 16 to 25 years old; (3). 26-35 years old; (4) 36 to 45 years old; (5). 46-65 years old;

(6) Over 65 years old;

4. Occupation is: (1) Student; (2) Worker;(3) Staff; (4) Civil Servant; (6) Teacher; (7) The Self-Employed; (8) Farmer; (9) Military;

(10) [Retiree](javascript:;); (10) Others;

5. Your main way of travelling on weekdays: (1) Bus; (2) Rail transit; (3)Taxi; (4) Cars; (5) [Electric](javascript:;) Bicycle; (6) Bicycle; (7) Walking;

6. The purpose of your trip: (1) Work;(2) school;(3) Business; (3)Shopping; (4)Entertainment and sports; (5)Visit relatives and friends; (6) Hospital; (7) Home;

7. The average number of times you take a bus per week is: (1)more than 15 times; (2) 10 to 15 times; (3) 5-9 times;(4) 0 to 4 times;

8. Your current monthly salary: (1) <1650 RMB; (2) 1651-2500 RMB; (3) 2501-4077 RMB; (4) 4078-6000 RMB; (5) 6001-10000 RMB;(6) > 10000 RMB;

9. Vehicle situation in your family: (1) have many cars; (2)There is a car; (3) [Electric](javascript:;) Bicycle; (4) The bicycle;(5) None;

1. **Waiting time**

10. Your usual waiting time at the station is about:

(1) 0-5 minutes; (2)6-10 minutes; (3) 11-15 minutes;(4) 16-20 minutes; (5) More than 20 minutes;

11. Your satisfaction with the waiting time: (1) Happy; (2)Satisfied; (3) Basically satisfied; (4)Not satisfied;

12. Your satisfaction with the punctuality of the first and last bus: (1) Happy; (2)Satisfied; (3) Basically satisfied; (4)Not satisfied;

1. **Transfer convenience**

13. How do you feel about the current bus route? (1) Happy; (2)Satisfied; (3) Basically satisfied; (4)Not satisfied;

14. How do you think of the current transfer convenience between bus and bus?

(1) Happy; (2)Satisfied; (3) Basically satisfied; (4)Not satisfied;

15. How do you feel about the current transfer convenience between bus and rail transit?

(1) Happy; (2)Satisfied; (3) Basically satisfied; (4)Not satisfied;

16. How do you feel about the current transfer convenience between bus and bicycle?

(1) Happy; (2)Satisfied; (3) Basically satisfied; (4)Not satisfied;

1. **Service attitude**

17. How do you feel about voice announcement in the bus? (1) Happy; (2)Satisfied; (3) Basically satisfied; (4)Not satisfied;

18. Are you satisfied with the service attitude of the company and crew? (1) Happy; (2)Satisfied; (3) Basically satisfied; (4)Not satisfied;

19. Are you satisfied with the use of air conditioning in accordance with the regulations?

(1) Happy; (2)Satisfied; (3) Basically satisfied; (4)Not satisfied;

20. Are you satisfied with the fare discount for the aged, students and IC cards?

(1) Happy; (2)Satisfied; (3) Basically satisfied; (4)Not satisfied;

1. **Travel information service**

21. Are you satisfied with the clarity of the station nameplate? (1) Happy; (2)Satisfied; (3) Basically satisfied; (4)Not satisfied;

22. Are you satisfied with the signs (such as route, warning signs, service and complaint phone number, etc.) ? (1) Happy; (2)Satisfied; (3) Basically satisfied; (4)Not satisfied;

23. How do you feel about the public transport information service (public transport information query online and on APP of mobile phone)? (1) Happy; (2)Satisfied; (3) Basically satisfied; (4)Not satisfied;

24. How convenient it is for you to recharge your IC card? (1) Happy; (2)Satisfied; (3) Basically satisfied; (4)Not satisfied;

1. **Ride comfort**

25. How do you feel about the new and old performance of the vehicle and the safety of the seat and armrest of the vehicle? (1) Happy; (2)Satisfied; (3) Basically satisfied; (4)Not satisfied;

26. How do you think the bus is crowded during the rush hour? (1) Happy; (2)Satisfied; (3) Basically satisfied; (4)Not satisfied;

1. **Waiting environment**

27. How do you feel about the waiting facilities at the intermediate stations?

(1) Happy; (2)Satisfied; (3) Basically satisfied; (4)Not satisfied;

28. How do you feel about the waiting facilities and convenience facilities at the terminal stations?

(1) Happy; (2)Satisfied; (3) Basically satisfied; (4)Not satisfied;

1. **The sanitation environment**

29. How do you feel about the sanitation condition of the bus? (1) Happy; (2)Satisfied; (3) Basically satisfied; (4)Not satisfied;

30. How do you feel about the sanitation facilities in the car? (1) Happy; (2)Satisfied; (3) Basically satisfied; (4)Not satisfied;

**Number**___ _____**Investigator**______ __

**Passenger Satisfaction Questionnaire of public Transport System in Ningbo City – Rail transit**

Dear Madam/Sir,

We are researchers of urban traffic planning in Zhejiang Normal University, the purpose of this survey is to promote the construction of "Ningbo transit city". Thank you for taking time out of your busy schedule to fill in this questionnaire. The investigation will be conducted anonymously, and we will strictly abide by the relevant provisions of the State Secrets Law. Thank you for your help!

College of Engineering, Zhejiang Normal University

**Instructions: Please tick the serial number you think is appropriate. The questionnaire is single choice without special instructions.**

1. **Basic information**

1. Your current rail transit line:________

2. Gender: (1) Male; (2) female;

3. Age: (1) under 15 years old; (2) 16 to 25 years old; (3). 26-35 years old; (4) 36 to 45 years old; (5). 46-65 years old;

(6) Over 65 years old;

4.Occupation is: (1) Student; (2) Worker;(3) Staff; (4) Civil Servant; (6) Teacher; (7) The Self-Employed; (8) Farmer; (9) Military;

(10) [Retiree](javascript:;); (10) Others;

5. Your main way of travelling on weekdays: (1) Bus; (2) Rail transit; (3)Taxi; (4) Cars; (5) [Electric](javascript:;) Bicycle; (6) Bicycle; (7) Walking;

6. The purpose of your trip: (1) Work;(2) school;(3) Business; (3)Shopping; (4)Entertainment and sports; (5)Visit relatives and friends; (6) Hospital; (7) Home;

7. The average number of times you take a bus per week is: (1)more than 15 times; (2) 10 to 15 times; (3) 5-9 times;(4) 0 to 4 times;

8. Your current monthly salary: (1) <1650 RMB; (2) 1651-2500 RMB; (3) 2501-4077 RMB; (4) 4078-6000 RMB; (5) 6001-10000 RMB;(6) > 10000 RMB;

9. Vehicle situation in your family: (1) have many cars; (2)There is a car; (3) [Electric](javascript:;) Bicycle; (4) The bicycle;(5) None;

1. **Waiting time**

10. Your usual waiting time at the station is about:

(1) 0-5 minutes; (2)6-10 minutes; (3) 11-15 minutes;(4) 16-20 minutes; (5) More than 20 minutes;

11. Your satisfaction with the waiting time: (1) Happy; (2)Satisfied; (3) Basically satisfied; (4)Not satisfied;

12. Your satisfaction with the punctuality of the first and last bus: (1) Happy; (2)Satisfied; (3) Basically satisfied; (4)Not satisfied;

1. **Transfer convenience**

13. How do you feel about the time of the last train in the current rail transit?

(1) Happy; (2)Satisfied; (3) Basically satisfied; (4)Not satisfied;

14. How do you feel about the current rail transit frequency during rush hour?

(1) Happy; (2)Satisfied; (3) Basically satisfied; (4)Not satisfied;

15. How do you feel about the current transfer convenience between bus and rail transit?

(1) Happy; (2)Satisfied; (3) Basically satisfied; (4)Not satisfied;

16. How do you feel about the current transfer convenience between rail transit and bicycle?

(1) Happy; (2)Satisfied; (3) Basically satisfied; (4)Not satisfied;

1. **Service attitude**

17. How do you feel about the current transfer convenience between rail transit and private car?

(1) Happy; (2)Satisfied; (3) Basically satisfied; (4)Not satisfied;

18. How do you feel about the active service of the rail transit staff and the staff's response to inquiries, consultations, complaints?

(1) Happy; (2)Satisfied; (3) Basically satisfied; (4)Not satisfied;

19. Are you satisfied with the convenience of using the automatic ticket machine?

(1) Happy; (2)Satisfied; (3) Basically satisfied; (4)Not satisfied;

20. Are you satisfied with the layout of the check-in machine of the station.

(1) Happy; (2)Satisfied; (3) Basically satisfied; (4)Not satisfied;

1. **Travel information service**

21. How do you feel about the promptness of informing passengers about emergencies?

(1) Happy; (2)Satisfied; (3) Basically satisfied; (4)Not satisfied;

22. How do you feel about the information service of rail transit and the layout guide signs?

(1) Happy; (2)Satisfied; (3) Basically satisfied; (4)Not satisfied;

23. How do you feel about the information service of rail transit (information query online and mobile APP) ? (1) Happy; (2)Satisfied; (3) Basically satisfied; (4)Not satisfied;

24. How do you feel about the calls quality of mobile phone in rail transit?

(1) Happy; (2)Satisfied; (3) Basically satisfied; (4)Not satisfied;

1. **Ride comfort**

25. How do you feel about the interior air and the comfort of air conditioner, seat of the vehicle?

(1) Happy; (2)Satisfied; (3) Basically satisfied; (4)Not satisfied;

26. How do you think the rail transit is crowded during the rush hour? (1) Happy; (2)Satisfied; (3) Basically satisfied; (4)Not satisfied;

1. **Waiting environment**

27. Are you satisfied with the accessibility and cleanliness of the station?

(1) Happy; (2)Satisfied; (3) Basically satisfied; (4)Not satisfied;

28. How do you feel about the waiting facilities and convenience facilities at the terminal stations?

(1) Happy; (2)Satisfied; (3) Basically satisfied; (4)Not satisfied;

1. **The sanitation environment**

29. How do you feel about the cleanliness and hygiene of the train? (1) Happy; (2)Satisfied; (3) Basically satisfied; (4)Not satisfied;

30. How do you feel about the aesthetics of the internal environment of the train?

(1) Happy; (2)Satisfied; (3) Basically satisfied; (4)Not satisfied;

**Number**___ _____**Investigator**______ __
